# From Model to Everyday Practice: A Qualitative Observational Study of Daily Fact Team Board Meetings

**DOI:** 10.5334/ijic.7555

**Published:** 2023-10-10

**Authors:** Ingunn Myraunet, Anita Strøm, Heidi Moen Gjersøe

**Affiliations:** 1 VID Specialized University, Oslo, Postbox 184, Vinderen, 0319 Oslo, NO

**Keywords:** mental health, interprofessional collaboration, FACT, health services, integrated care

## Abstract

**Introduction:**

The Flexible Assertive Community Treatment (FACT) model has rapidly become a way of organising services for people with severe mental illness. FACT describes the integrated approach of interprofessional teams.

**Method:**

A qualitative study of interprofessional collaboration in three FACT teams was conducted. Thirty observations of the teams’ board meetings were conducted, and field notes were thematically analysed.

**Results:**

This study generated three themes in interprofessional collaboration in FACT teams. The first theme reflects the challenges of working in line with the model, the second suggests an unclear understanding of a shared caseload, and the third shows different approaches to working with a shared caseload.

**Discussion:**

The themes suggest that there is increased opportunity for the shared caseload in the FACT team board meeting. The findings reflect that there is a lack of either the resources necessary for working with a shared caseload or an understanding of the intention of a shared caseload.

**Conclusion:**

The potential of the shared caseload in FACT team board meetings are dependent on sufficient resources and a collective understanding of the FACT model and the shared caseload among professionals. Further research on how a shared caseload is experienced and facilitated in FACT teams can provide insight into their practices.

## Introduction

Interprofessional collaboration is a widespread way of organising healthcare services to meet the needs of society. Thus, the places and arenas where health- and social care professionals (hereafter called professionals) conduct their work have changed [1]. Studies indicate that working in an interprofessional team can lead to a redistribution of roles, which can be associated with changes in the identities of the involved actors and the assimilation of professions [2]. The barriers to team collaboration are mostly associated with a lack of knowledge and awareness about other professionals’ competencies and roles, the sharing of information, and responsibilities [3].

The Flexible Assertive Community Treatment (FACT) model has rapidly gained ground as a recovery-oriented model whereby professionals work with a shared caseload to support people with severe mental illness [4,5]. The shared caseload entails having a variety of professionals on the team to support patients’ complex needs [6]. The teams have a FACT team board meeting daily, or at least three times a week, to engage in collective discussions around patient care and to share their views and knowledge [7]. The literature suggests that barriers to collaboration are related to different professional boundaries, backgrounds, and hierarchy [8]. There is scarce evidence of the different contributions of professionals within teams, and the effects of these are indicative [3]. For these reasons, interprofessional teams constitute a complex working environment for professionals.

This study contributes to the research concerning how professionals conduct their collaboration in daily FACT team board meetings. Implementing FACT teams has been proven to be quick and easy in other Scandinavian countries [9], and often on a large scale, such as in Norway [4,10]. Other studies have found positive attitudes among professionals towards working together in a flexible way and evidence that FACT enhances the care given to patients [5]. However, how the team members in the FACT model collaborate and work with a shared caseload in their daily board meetings has received less attention in research. Therefore, this issue will be highlighted in the present study by asking: How is the FACT model’s shared caseload practised in daily FACT team board meetings?

## Background

### Interprofessional collaboration

Interprofessional collaboration can be defined as “something more than the simple sum of its parts […] this implies a high level of communication, mutual planning, collective decisions, and shared responsibilities” [11]. Collaboration in an interprofessional team has been found to protect people from burnout due to team support [12]. The factors supporting collaboration in interprofessional teams include respectful communication between disciplines, regular monitoring, and evaluation to improve the practice [13]. Daily meetings can improve interprofessional collaboration by reminding professionals of their overall goal, which is – to improve their patients’ health and well-being [14]. Furthermore, daily meetings foster trust in the workplace and bridge communication barriers between various professional groups [14]. In their systematic review, Schot, Tummers, and Noordegraaf [3] state that interprofessional teams can close professional, social, physical, and task-related gaps, negotiate roles and tasks in collaboration, and make space for collaboration. The main weakness of the research on interprofessional collaboration is that it mostly concerns the experience of doctors or nurses [3] Research is scarce, however, regarding the differences in various approaches and the effect of the different contributions of professionals within the teamwork [3]. Moreover, interprofessional collaboration is limited when a positive work environment, and the integration of knowledge from the different disciplines involved are lacking [15]. The vision of integrated care is promising, but it remains a complex phenomenon, and the evidence of positive outcomes is mixed because the views, interests, and objectives among the actors differ [16]. Thus, studies emphasise that teams must have common sensemaking to create shared understanding [17]. Establishing integrated care is a multifaceted and long-term process, and a review of integrated care found difficulties in showcasing the causality between the delivery of integrated care and outcomes [18].

Studies from Australia, Ireland, New Zealand, Sweden, the United Kingdom, and the United States have pointed out that interprofessional teams within the mental health service context have received limited guidance on collaboration, compared to other multidisciplinary teams (e.g. cancer care) and would benefit from reviewing the practices in other fields of health [13].

### Flexible assertive community treatment

**Figure 1:**
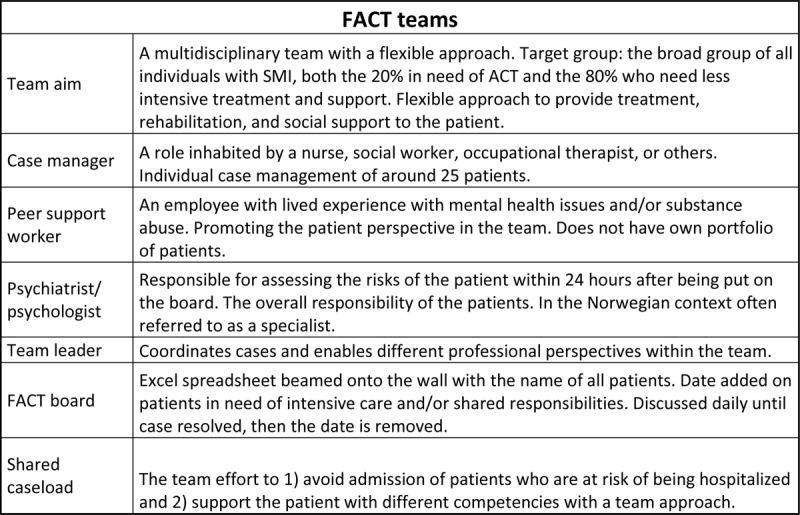
Information about FACT teams based on Veldhuizen and Bähler [6].

Most resources aimed at benefitting people with severe mental health issues have traditionally been directed towards hospital treatment [1]. However, the treatment of mental illness has changed considerably during the past 50 years due to the de-institutionalisation of mental health services [1,19,20]. In addition, the complexity of the needs of people with severe mental illness has prompted the development and implementation of different models to support these needs through interprofessional teamwork [19,20]. Nowadays, the treatment of persons with mental illness is primarily conducted in the areas where people live [19,20]. Stein and Test’s Assertive Community Treatment (ACT) model focuses on the most vulnerable 20% of people with severe mental illnesses [21], and it is the most studied case management model [22]. The Flexible Assertive Community Treatment (FACT) model, see Figure 1 for details, builds on ACT, and both are rehabilitation-oriented clinical case management models. However, FACT includes a broader range of clients with severe mental health issues [23]. The model was initially developed in the Netherlands [24] but is now a widespread way of supporting the recovery-oriented processes of clients with mental illness in Belgium, Hong Kong, the United Kingdom [6], Denmark [25], Norway [4,10,26], and Sweden [5,9]. The FACT fidelity scale has developed from mainly using quantitative measurements to focusing more on qualitative measures that ensure recovery-oriented treatment [23]. The 2010 fidelity scale has been helpful for new teams to implement the FACT model properly [27].

Interprofessional FACT teams’ valuing of the different perspectives of professionals supports their goals of maintaining continuity in their patients’ care and avoiding hospital admission [24]. The shared caseload provides a common action space, and professionals report enjoying this way of working because it reduces job strain and stress and increases control in their workdays [5]. The concept of a shared caseload offers teams the ability to increase the intensity of care together [27]. A FACT team is integrated which means that the team members have different areas of expertise and can divide among themselves the interventions their clients need, so there is little need for the involvement of other teams or services. The team coordinates the interventions on a FACT team board and meet regularly so that everyone involved understands the approaches to be taken. Clients on the FACT team board are discussed daily until their issues are resolved. The team approach provides clients with treatment and intensive care from multiple or all team members [6]. During a FACT team board meeting, the clients in need of intensive care, i.e., the 20% who need ACT, are discussed to determine the – what, when, and how factors in providing support and care, and they are visited daily or more often. The 80% of clients with less need for intensive care receive individual case management, a role that can be assumed by all team members from various of disciplines. The FACT team board meeting ensures that the team knows which clients are included in the different groups, and the switching mechanism between individual case management and intensive support is the essential element of FACT [6]. The case manager is the first contact person of the client, who visits and monitors the client and makes sure the treatment plan is up to date. Every client has a second case manager who is up to date on treatment and can take over immediately. In times of less intensive care, the case manager requests short-term interventions from other disciplines [6]. The team’s psychiatrist or psychologist is responsible for the risk assessment of all the clients and is involved in making the treatment plans. The team leader’s role is to facilitate cooperation and coordination between the professionals involved in the FACT team [6].

Several positive aspects of the FACT model have been identified [5], and professionals working within FACT teams have reported positive attitudes towards interprofessional collaboration [5,28]. Implementing FACT teams is also perceived to be relatively quick and easy, and the professionals positive attitudes towards FACT are an essential factor in its implementation [9].Nielsen [25] investigated practitioners’ experiences when transitioning from working either in an ACT team or community mental health team (CMHT) to working in a FACT team. Their study found that the CMHT practitioners had a more positive experience with the shared caseload, as the ACT practitioners were more worried that hard-to-reach patients would receive less support due to having an increased caseload [25]. Trane et al. [4] explained that FACT teams close several gaps in the fragmented services, but that the complexity of the service can hamper the functioning of FACT teams [4]. In another study, Trane and Aasbrenn investigate how the service system creates challenges for collaboration [26], especially in rural areas [10]. On a service delivery level, the model’s flexibility and professional freedom are perceived to enhance the care provided to clients [5].

Moreover, studies indicate that the structure of the FACT model provides professionals with a precise aim and leads to good client treatment [29]. In this way, the model can provide guidance, direction, and flexibility to interprofessional teams and offer a promising way to organise mental health services. However, studies underline a lack of research concerning how the professionals in FACT teams collaborate and how the professionals’ different backgrounds contribute to the collaboration [24]. This article aims to contribute to the discussion concerning the interprofessional collaboration in FACT teams by investigating how professionals work together in team board meetings.

## Method

The qualitative method is useful when studying issues in their natural settings and attempting to make sense of, or interpret, phenomena according to the meanings people bring to them [30,31]. Observation is a key tool for collecting data within qualitative studies [30]. Insight can be gained by observing the physical settings, participants, activities, interactions, conversations, and the researchers one’s role [30]. This study’s observational approach was chosen to better understand how teams interact during FACT team board meetings, and how they work with a shared caseload in the FACT model.

### Role of the researcher

Research is shaped and situated by the researcher conducting it [32]. As this study was conducted at a place previously known to the researcher, an account of the pre-understandings and prejudices was written down to increase awareness of potential cultural blind spots. The researcher had work experience in the field and was familiar with the practical arrangement of the digital FACT board, the patient journal system, and the team’s abbreviations. Observations of body language, verbal communication, and thoughts/reflections were written down in the field notes while observing the teams. Creswell and Poth [32] describe four different approaches and emphasise that the observation role can shift from complete participant to complete observer. During the observations, partial participation was chosen as an approach [33]. This means taking on the role of “participant as observer” [34] before and after the team meetings, wherein the researcher interacted with the team members. This approach allowed the researcher to gain insight into the insider view and subjective data [30]. When the FACT team board meeting started, the role shifted to “observer as participant,” in which the researcher did not participate and focused on taking notes and watching the team interaction without involvement [34]. This approach allowed the researcher to be a more natural part of the environment, interacting with the team members before and after the meeting, without verbally disturbing the teams’ daily discussions during the meeting [30]. The field notes were taken by hand, and direct quotes from the team members were written down. The decision to write by hand and not computer was to minimise the noise in the meeting. Due to sensitive client data, audio recordings or videos were not possible. After the observations, thorough situational notes were written out on computer, which did not include specific patient information or diagnosis.

### Selection and data collection

The recruitment of teams was done by reaching out to the steering group of FACT teams and recruiting teams that had been operational for over one year, which in turn introduced another FACT steering group. The steering groups granted their permission to conduct the study, and the study’s aim of was presented to the teams, who voiced their desire to participate. Three Norwegian FACT teams were included. They were based in an urban area, and worked according to the 2010 fidelity scale. The teams served either the traditional FACT team population, specialised groups within mental health or people with substance use disorder. The scope of observation was the daily FACT team board meetings, which were sometimes extended due to discussions of caseloads or treatment plans. This setting was chosen due to the meeting’s goal of discussing the shared caseload. Ten physical observations were conducted in each of the three teams, totaling 30 observations. Due to the Covid-19 restrictions, employees with symptoms were required to stay home until they showed a negative test. Therefore, some meetings had only three members physically present while the other team members participated digitally. Furthermore, they were expected to cover other team members caseload during this time, so the actual work of each case manager was not clearly defined. The observations lasted 30–90 minutes and were carried out between September and November 2021.

#### Team description

The teams consisted of ten employees with various caseloads, and each team had a designated team leader and peer support worker. The teams often referred to psychiatrists and psychologists as “specialists,” and these professions had reduced caseloads and responsibility for risk assessment of all patient. Other professionals were referred to as case managers. Depending on the team composition (see Table 1 for more information), there were a variation of between four and five case managers in each team and two to four specialists. The teams differed in how long they had worked together, the professions represented, and the number of patients they served. All teams had moderate to satisfactory fidelity and worked with adults 18 years + with severe mental illness and/or substance use disorder. The FACT team’s patients were listed on the FACT board, with a date beside the names that needed to be discussed. When a client was considered to only need individual case management, the date beside the client’s name was removed from the FACT team board.

**Table 1:** Team members in three teams.


ROLES IN THE TEAM	NUMBER EMPLOYEES

Team leader and case manager	3

Psychiatrist/psychologist responsible for risk assessment	9

Case manager, nurses	6

Case manager, health or social care professionals	7

Peer support worker	3

Employment specialist	2

Total	30


### Data analysis

Reflexive thematic analysis was applied to interpret the material [35]. The researcher read the data thoroughly multiple times to familiarise with the material and wrote reflection notes along the way. These first rounds intended to create a view of the interactions during the observations. N-Vivo was then used to generate the first codes and initial interpretations of the observational data material. The initial codes were discussed with the second and third authors, and the themes were presented at a descriptive level. By going back and forth between the material, a hermeneutic approach was taken towards the text as a whole and the singular observations in the different contexts, with the aim of understanding the field notes within a broader context [36]. In line with this approach, all the descriptive themes were written on paper and arranged into groups which developed into preliminary themes across the entire dataset. These themes were discussed with the second and third authors, who have expertise in qualitative methods. They made the contradictions within each theme more explicit, thus indicating the need to write more precisely. Finally, the material was examined again, creating a mind map to better understand get a better view of the relations between the themes better, and describe in depth what each theme entailed. A map of this analysis was formed by checking the themes in relation to the coded material and the dataset as a whole. The issues of lack of resources, change of staff, variation in FACT training and unclear understanding of the model, creates the theme that suggests challenges with working according to the model. The uncertainty of who decides, when to pull others in, and coordinate support contributed to an unclear understanding of the shared caseload. Collaborations, relationships, trust, and knowledge of each other’s formal and informal expertise reflected the teams’ different approaches to the shared caseload.

### Ethics and consent

The Norwegian Centre approved the project for Research Data (NSD) nr 928818, and the researcher was granted an exception from the non-disclosure agreement by the Regional Committees for Medical and Health Research Ethics (REK) nr 268065. The project was presented and approved by the FACT steering groups. The purpose of the study was explained to each team, participation was voluntary, and they were allowed to ask questions. Written consent to observe the team was obtained from all team members. The information provided has been handled confidentially and presented anonymously in line with the institution’s internal guidelines.

## Results

Based on the analysis, the authors developed the following three themes:

- Challenges to working in line with the model
- Unclear understanding of the shared caseload
- Different approaches to the shared caseload

### Challenges to working in line with the model

A shared understanding of what the team should do differed between the teams. Two teams clearly understood the elements of what FACT should offer but had difficulties due to a lack of or change of staff. The third team was focused on the theoretical understanding of the model. In the following example, a team with a shared understanding discusses what to do.

“The case manager gives an account of a patient who has been perceived as increasingly worsening in the last weeks and now needs more intensive support. After the team discussion, there is a consensus that the patient needs ACT support. First, the team leader says that it might be difficult to manage an ACT approach due to the lack of team members, as many are sick this week. Next, the team leader asks if anyone has the time. One team member says that Friday (two days from now) is open, but the patient is not familiar to them. Another case manager checks the calendar, and they decide to go together.” (Field notes)

The lack of staff in two teams seems to hamper the possibility of working according to the model and ACT principles. In this case, the team members have a common understanding of what is needed when there is an ACT request but lack the resources to follow the ACT principles. Even though the team does not know the patient, they coordinate their recourses to intensify the support. On several occasions, the teams talk about how they can work together and what support they can provide to patients. The members of the third team seemed to have different perceptions about the aim of the model, as they had recurring conversations about how to understand the model. As a result, there was uncertainty concerning a shared understanding of what the team should do.

“Today, the team had another conversation in which they wondered what perspective to prioritise in the treatment. There was a discussion about whether they should focus on a function- or recovery-perspective. This topic occurs quite often, as the team members have different views on how to support patients. One of the specialists asked, “Should we move forward with the case manager’s or the specialist’s idea?” One of the other team members reflected that the case manager needs to believe in the chosen treatment for the patient. Otherwise, there cannot be proper engagement from the case manager to make the treatment work. They seem to struggle to agree upon a course of action and ask themselves who should decide as if there is one solution to the question. The different ideas originate from the patient’s wishes, as stated in the treatment plan. In the end, the team leader draws a line by stating that the meeting is for discussing different solutions with all the different professional contributions. However, the case manager must choose and implement the treatment plan.” (Field notes)

In this team, there seems to be uncertainty about what FACT should offer their patients and how they should agree upon a course of action. However, rather than basing where to start on the patient’s needs, they discuss which approach and goal the team should choose to work with first. It seems that they have different perspectives and struggle to find a shared understanding of what to do.

### Unclear understanding of the shared caseload

The findings indicated that the teams had difficulties concerning the shared caseloads and how to contribute with their different professional reasonings in support of the patients. One team debated whether they should decide on a matter before a certain team member was back at work.

“There are a range of dilemmas in which the team members must make decisions. For example, today, a query from a patient’s network was directed to one of the team members who was not present. There were several aspects of the case that the team had to take into consideration. However, one of the specialists wondered if it was something they as a team should decide on or if they should let the missing team member handle the request upon return. The discussion continued, and in the end, they agreed that the team should decide and that they would give the information to the person who requested the contact and thus resolve the case before the member returns.” (Field notes)

The team discussed the case at length, going back and forth as to whether the team members could handle the situation alone or if they should do it as a team. As the case was not urgent in terms of patient care, it could have waited. However, because of the ethical dilemmas involved in the query, they decided that a member with thorough knowledge of the regulations and law would make the contact and explain why the query was rejected. This time, they decided to act on the case during their discussion. In another team, there was some confusion about the sharing of caseloads as the question of who should decide arose.

“The case manager had been working with a patient alone for some time and wanted input from the team members. They discussed what to do next. After some time with different inputs, the case manager who had met with the patient asked, “Who decides what we should do?” There seemed to be some confusion about how they should move forward, but the team leader answered that the team decides together what they should do. The case manager expressed a feeling of being alone in this case for over a year and said that it would be nice if some of the others could be involved. As some of the other team members had been involved in the beginning, they decided that the case manager would remind the patient of the shared caseload in FACT and that other team members would make contact as well.*”* (Field notes)

In this team, the case manager has provided individual case management and seems unsure when to request help from the team. The concept of a shared caseload does not seem to be understood and leads to uncertainty about who should decide what to do. The team leader supports the case manager plans so the case manager receives support from the team.

### Different approaches to the shared caseload

The teams’ ability to apply the various team members’ expertise differed. One team seemed to know each other quite well and recognised each other’s formal and informal competencies. In another team, there was an openness to ask for help or an explicit request to contribute with one’s perspective. In the team where they knew each other, they could pull each other in by making specific requests. As shown in the following meeting:

“Today’s meeting was less structured, as some team members had to leave early, but they gave an account of the clients they had visited before leaving. Afterward, the team circled back to the top of the FACT board updating patient information, coordinating support, and discussing as a team what to do. When a case manager presents a challenge regarding a patient, all members are attentive. The case manager can easily make eye contact with others during the presentation and request others to assist. It is interesting how the case manager addresses the other team members’ formal and informal competence, such as asking the social worker to assist with some welfare issues and requesting that the specialist look at the patient’s PlayStation. They must know each other quite well in this team. The team members are positive in contributing with their expertise, and the case manager expresses that having more people to support the patient is nice, as they can work together to solve the issues at hand.” (Field notes)

In this team, the members appeared to know each other quite well and asked directly for assistance in addressing issues. There seemed to be less knowledge of the team expertise in another team. Nevertheless, they also asked for assistance and if someone had the knowledge to support a patient.

“During the FACT board meeting, the case manager asked for advice on what to do when a patient did not get up until the late afternoon and therefore had trouble meeting with the case manager. A specialist suggested that they needed to work on the patient’s sleep habits. The case manager queried how to do that and asked the specialist, “Can you help me with it?” The specialist replied positively to the request, and they coordinated how they could work together with the patient.” (Field notes)

In this situation, the case manager requested suggestions on how to reach the patient and help implement a solution. The case manager and specialist decided work together to help the patient back on track with the sleep pattern.

However, in the last team, there was a different approach to involving the various perspectives of the team members. As they had different perspectives on what working with a shared caseload meant, the professionals in this team tended to give isolated support, based on their respective professional backgrounds. In a discussion about working with a patient where there had been some challenges collaborating with the next of kin, and the team discussed what to do and whether more people should be involved.

One of the case says, “It’s not like he [the specialist] is the therapist; we are a team, but right now, we are working as an outpatient clinic where he [the patient] can change therapist. But we are supposed to be one voice, the whole team.” As the team continues to discuss a treatment plan, there is a wish from one of the case managers to make the plan less alienating for the patients and that they would have liked to have their own “dummy treatment plan,” not the one they have adopted from the hospital. Another case manager says “If we were a team, we could have investigated this a bit more. Made a plan to visualize how we as a team are working with the patient.” A third case manager agrees, “Yeah, that would have been fun, to see how we can work towards the patient’s goal, discuss it in the team and see what direction to go.” A specialist joins in, “Yes, to be connected from the beginning.” Another case manager continues, “So that we could actually use each other and work with coping mechanisms and recovery… what we really should be doing.” The specialist says, “To have more focus on what we can do, how we can help, and start there. That would have been nice to try”. (Field notes)

In this conversation, the team reflects on their own practice and that they work as individual professionals, not as a team with a shared caseload.

## Discussion

Interprofessional collaboration requires a high level of communication [37] hence the daily board meetings in FACT, where the team members can coordinate their work and share the patients’ needs, wishes, and goals [27]. These meetings give the team members the opportunity to share the overall goals of treatment and the patients’ wishes [14]. An evaluation of FACT in Norway highlighted that the professionals involved had positive experiences with the model. The daily FACT team board meeting were essential for maintaining the structure, shared goals, and responsibilities [29]. Nevertheless, while the teams share information in the daily board meetings there is a variation between the teams’ practices. Even though there have been developed guidelines for FACT team board meetings [7] the findings in this study suggest that the guidelines are not yet fully implemented. This indicates that even though teams implement the FACT models quantitative fidelity scale from 2010, integrating knowledge and a shared caseload in board meetings is challenging. Either because of the lack of recourses or different understanding of the FACT models shared caseload. There are, however, some indications that new FACT teams can profit from using the 2010 fidelity scale [27]. As most of the professionals in these teams are experienced practitioners in mental health, they may have benefitted from having more reflection around the model, as the R2017 fidelity scale provides.

This study emphasis the importance of the teams’ needs to have sufficient resources, and a common understanding of the model, and to share caseloads to benefit from the model. Teams lacking resources struggled to meet the ACT criteria. Because the teams lacked members, some members experienced increased workload, which interfered with their workday flexibility. As flexibility is highlighted as a core element [27], that professionals’ value in the model [5], it is crucial that this feature is present in their workday. A lack of resources over time might hinder the team support, a support which has also been highlighted as positive in teamwork [15]. In addition, a lack of resources might indicate that the team has experienced challenges that have led to burnout [12], which in turn creates a greater lack of resources.

An evaluation of FACT teams found that interprofessional teams were viewed as positive [29], possibly due to the sharing of disciplinary expertise and the application of knowledge across different contexts [38]. The findings of this study related to the teams’ understanding of the FACT model and shared caseload suggests variations between the teams. One team is debating whether they should prioritise therapy or recovery, this might indicate that they have not reached a consensus about how to work with the FACT models shared caseload. Perhaps they cannot integrate each other’s different expertise [37] and combine that of the team members in a new organisational model [38], in which case, they might miss out on the benefit of a shared caseload. They could have achieved these aims by agreeing on who works with which of the patients’ wishes and how. Supporting several of the patient’s needs [27], instead of settling for one perspective. From another angle, one might question whether they have understood the recovery orientation in the FACT model or if they are working more like therapists with individual clients. If there is more weight given to providing individual therapy, one might question if the team has had enough training in the FACT principles and is familiar with the concept of the shared caseload. Therefore, a possible benefit of adhering to the R2017 fidelity scale, which focuses more on qualitative, reflexive practice, is that the team might realise an increased integration of each other’s expertise.

Concerning the shared caseload, one team works together to contribute solutions, but the case manager decides which goals to prioritise. This can be related to a lack of understanding of how to work with the shared caseload as a team. A crucial factor in interprofessional collaboration is having a common aim in the team, which Allred, Burns, and Philips [17] describe as creating a shared understanding. If there is no common ground from which the team members interact, there can be a problem with integrating different professional perspectives [37]. Nevertheless, by focusing on a shared caseload and working accordingly, integrating each other’s knowledge might be implemented more in the FACT teams’ board meetings. Hence, it might be helpful to know if the employees in the different teams have had the time to complete proper training and if they were optimistic about working with the model beforehand, which is one of the significant findings in Svensson et al. [9].

Another finding in this study suggests that when the professionals who recognise each other’s competencies can access the shared caseload more easily. In a team where the members know each other well, this is accomplished by directing their requests to specific team members. Teams with less knowledge of other team members, can still ask if anybody has the required knowledge to contribute. During the discussions, it is often the case manager who either asks for input from the team or to specific team members. This might be due to the more traditional way of case management method, in which team members are responsible for their own cases.

### Strengths and limitations

Observation as a method offers the possibility of seeing how the teams work with the aim of a shared caseload in the FACT team board meetings. In this study, the reason for observing the team instead of interviewing the team members was to see how they practised a shared caseload, as this is what other studies have highlighted as a positive element of the FACT model [5]. The approach might have been strengthened if recordings had been allowed at the meetings to facilitate the conversations with the other authors who were not present at the board meetings. However, due to the restrictions of the REK, this was not a possibility. Furthermore, to validate the findings the researcher could have revisited the teams to observe further developments, and discuss findings with team members. In this study, the authors discussed the different possibilities, and decided that 30 observations were sufficient to achieve the research aim - to gain knowledge of how shared caseload is practiced in the daily FACT team board meetings. The interpretations of the first author were discussed at length to reduce the possibility of this individual’s pre-understandings reflecting on the themes.

## Conclusion

The findings of this study provide insight into the importance of having enough team resources, and team members taking the time to build a shared understanding of FACT, and an understanding of how they can work with a shared caseload. The FACT team board meeting provides professionals with an arena in which to focus on a shared caseload. However, a lack of resources decreases the flexibility of the team and their potential to work with a shared caseload, thus, increasing their focus on achieving quantitative measurements in the FACT fidelity scale. The findings of this research emphasise the importance of a shared understanding among the professionals in interprofessional teamwork. When implementing FACT in a new service system, there is the risk that a dynamic way of working with the model may be lost.

Furthermore, the importance of having a shared understanding is an important discussion when implementing new models and collaborative practices in existing services. The findings show that teams with knowledge about the formal and informal competence of team members are more likely to engage others’ expertise. In conclusion, this study contributes to the field of interprofessional collaboration by showing that open communication enables the management of shared caseloads that can benefit patients. Interprofessional teams might better realise the potential of the shared caseload during the FACT team board meetings by using the FACT team board meeting guidelines more actively and/or by adopting the R2017 fidelity scale. Further research on how team members experience collaboration within the team might give more insight into what competence is needed to actively work with the shared caseload in FACT teams.
